# Effects of Essential Amino Acid Deficiency on General Control Nonderepressible 2/Eukaryotic Initiation Factor 2 Signaling and Proteomic Changes in Primary Bovine Mammary Epithelial Cells

**DOI:** 10.3390/cimb44030071

**Published:** 2022-02-25

**Authors:** Zulma Tatiana Ruiz-Cortés, Peter Yoder, Mark D. Hanigan

**Affiliations:** 1 Research Group Biogénesis, Faculty of Agricultural Sciences, University of Antioquia, Medellín 050034, Colombia; 2 Perdue AgriBusiness, Technical Service Manager, Salisbury, MD 21804, USA; peter.yoder@perdue.com; 3 Department of Dairy Science, Virginia Tech, Blacksburg, VA 24061, USA; mhanigan@vt.edu

**Keywords:** caseins, eukaryotic initiation factor 2, protein biosynthesis, epithelial cells, homeostasis

## Abstract

We hypothesized that the general control nonderepressible 2 (GCN2)/eukaryotic initiation factor 2 (eIF2) signaling pathway and intracellular protein synthesis (PS) are regulated to maintain milk PS in primary bovine mammary epithelial cells (MECs) under essential amino acid (EAA) starvation conditions. We cultured MECs with 0%, 2% (depletion), and 100% (control) EAA for two exposure times (8 and 24 h), followed by three refeeding (RF) times with 100% EAA (0, 8, and 24 h). Subsequently, we measured cell viability, total protein concentration, and proliferation. Western blotting was used to quantify the levels of casein and the expression of total GCN2 and eIF2, as well as phosphorylated GCN2 (GCN2P) and eIF2 (eIF2P). The ISOQuant method was used to assess MEC proteomes, and the resultant data were analyzed using the Kruskal–Wallis test, nonpaired Wilcoxon rank post-hoc test, and ANOVA–Tukey test, as well as principal component analyses and multiple regressions models. Differences in cell viability were observed between the control versus the depleted and repleted MECs, respectively, where 97.2–99.8% viability indicated low cell death rates. Proliferation (range, 1.02–1.55 arbitrary units (AU)) was affected by starvation for 12 and 24 h and repletion for 24 h, but it was not increased compared with the control. Total protein expression was unaffected by both depletion and repletion treatments (median 3158 µg/mL). eIF2P expression was significantly increased (*p* < 0.05) after treatment with 2% EAA for 8 and 24 h compared with 2% EAA with 8 h + 24 h RF and 2% EAA with 24 h + 8 h RF. GCN2P also showed significantly increased expression (*p* < 0.05) after treatment with 2% EAA for 24 h compared with the control and 2% EAA with 24 h + 8 h RF. Intracellular casein/α-tubulin expression was unaffected by 2% EAA compared with control (0.073 ± 0.01 AU versus 0.086 ± 0.02 AU, respectively). We studied 30 of the detected 1180 proteins, 16 of which were differentially expressed in starved and refed MECs. Cells faced with EAA deficiency activated the GCN2P/eIF2P pathway, and the lack of change in the levels of casein and other milk proteins suggested that the EAA deficit was mitigated by metabolic flexibility to maintain homeostasis.

## 1. Introduction

Maintaining amino acid (AA) homeostasis is crucial for cell survival and normal cellular function, growth, and secretion. Because intracellular AA concentrations are dynamic, AA demand for protein synthesis (PS) must be matched with cellular AA uptake and supply (arising from protein degradation or from external supply). The catabolic process of autophagy is specifically activated in response to AA starvation via two key signaling cascades: the mammalian target of rapamycin (mTOR) complex 1 (mTORC1) pathway and the general control nonderepressible 2 (GCN2) pathway. These pathways are key regulators of the integration between anabolic (AA-depleting) and catabolic (AA-replenishing) processes that maintain intracellular AA homeostasis [1]. 

While mTORC1 responses are optimized to sense AA sufficiency, the GCN2/activating transcription factor 4 system in mammalian cells has evolved to sense AA restriction or, more precisely, AA imbalance among other processes [2]. The depletion of any AA will eventually result in uncharged tRNAs. These important signaling molecules bind to and activate protein kinase GCN2, which phosphorylates eukaryotic initiation factor (eIF) 2α. The latter can also be phosphorylated by other stress-activated protein kinases. In all cases, translation initiation is reduced, consequently reducing AA demand [3].

The rate of PS primarily depends on the rates of translation initiation and elongation, which are regulated by a number of eIFs and eukaryotic elongation factors (eEFs) [4]. Several essential AAs (EAAs) regulate the rate of casein synthesis by altering the phosphorylation state of intracellular signaling proteins that affect the activity of eIFs and eEFs [5]. Therefore, supplementation with specific AAs that exert the most influence on the pathways controlling eIFs and eEFs should reduce the amount of crude protein given to dairy animals, thereby increasing postabsorptive efficiency and reducing N excretion [6].

Recently, authors have illustrated the metabolic flexibility of the mammary gland in its use of aminogenic versus lipogenic substrates. The isoenergetic supplementation of aminogenic or lipogenic substrates modified the profile of AA and other metabolites available for mammary metabolism, and determined the level of mammary EAA uptake [7].

Prolactin signaling can regulate l-type amino acid transporter 1 (EAA transporter) and casein expression and activity in mammary epithelial cells of dairy cows, contributing to increased amino acid availability and milk protein synthesis in the mammary glands of dairy cows; the mechanism was recently studied and it was concluded that the signal transducer and activator of transcription 5 (STAT5) was the main transcription factor implicated [8] among the panoply of other proteins that could be interacting as reported in proteomic studies. 

The global proteome of bovine mammary epithelial cells (MECs) has been published [9], and various groups have performed proteomic studies of farm animals, including cows, with different levels of production at peak lactation [10]. The proteome dynamics of MECs isolated from lactating cows during different stages of lactation and from cows with different levels of milk production have also been reported [11,12]. However, information about the temporal responses of cells to varying nutrient supplies is scarce. Understanding the expression dynamics of various proteins in MECs will reveal how lactocytes respond to EAA deficiencies.

Elucidation of the relationship between AA homeostasis and mammary cell function is necessary to optimize milk PS and improve the AA metabolism efficiency. Thus, increased knowledge of PS regulation in MECs is critical to achieving a better understanding of quantitative AA requirements for milk PS.

In this study, we hypothesized that extracellular EAAs supplemented at varying concentrations and for varying exposure times would affect the cellular proteome, including the signaling proteins regulating PS, in bovine MECs. Accordingly, the objectives of this study were to measure the temporal changes in cell viability and proliferation, signaling changes in the GCN2/eIF2 pathway, and intracellular protein concentrations in response to EAA starvation and subsequent refeeding (RF).

## 2. Materials and Methods

### 2.1. Cell Culture and Treatments

MECs were obtained from the Chinese Academy of Agricultural Sciences [13,14]. The cells were seeded on six-well plates (Corning, Corning, NY, USA) and cultured in Dulbecco’s modified Eagle medium (DMEM/F-12; Gibco, Thermo Fisher Scientific, Waltham, MA, USA), to which glucose (3.15 g/L) and sodium bicarbonate (1.18 g/L) were added. The medium was supplemented with 50 mg/mL transferrin, 2 mg/mL insulin, 1 mg/mL prolactin, 25 mg/mL progesterone, 100 mg/mL hydrocortisone (Sigma, St. Louis, MO, USA), 0.25 mg/mL epidermal growth factor-EGF, 1% glutamine, 1% antibiotic–antimycotic mix (Sigma, St. Louis, MO, USA), and 10% fetal bovine serum (Gibco, Thermo Fisher Scientific). The cells were cultured for 6 days after reaching confluence (90–100%). Three treatment factors were assessed: nutritional level (0%, 2%, and 100% EAAs), fasting (depletion) time (8 and 24 h), and RF (repletion) time (0, 8, and 24 h).

The dependent variables were percentage viability, proliferation (density), total protein expression (µg/mL), casein levels (arbitrary unit, AU), total and phosphorylated GCN2 and eIF2 levels, and protein expression levels (proteome). Cells were verified to be of epithelial origin by immunohistochemistry using a pooled monoclonal antibody (MAb) AE1/AE3 (Thermo Fisher Scientific, Waltham, MA, USA) against epithelial markers, including cytokeratins. MAb AE1 recognizes keratins of the acidic subfamily with molecular weights of 56.5, 50, 50ʹ, 48, and 40 kDa, and AE3 MAb recognizes keratins with molecular weights of 65–67, 64, 59, 58, 56, and 52 kDa.

### 2.2. Cell Viability

Trypan blue solution (0.4%; Sigma, St. Louis, MO, USA), prepared in 0.81% NaCl and 0.06% K3PO4, was used to distinguish between live and dead cells. Briefly, the cells were released from the surface of the plates by trypsinization and suspended in the dye solution. Cell viability was determined by counting the average number of viable/live cells for each treatment in five fields of a hemocytometer.

### 2.3. Cell Proliferation

MECs were seeded in 96-well plates at an approximate density of 100,000 cells/well and incubated for 12–48 h according to the treatments. Subsequently, cell proliferation was assessed using the MTT colorimetric assay (Roche, New York, NY, USA). MTT is cleaved by metabolically active cells to form solubilized purple formazan crystals, and the optical density (OD) of the resulting colored solution is quantified spectrophotometrically.

### 2.4. Total Protein

MECs were washed twice in cold phosphate-buffered saline (PBS) and cold radioimmunoprecipitation assay (RIPA) buffer (Pierce, Thermo Fisher Scientific) containing three nonionic and ionic detergents. Then, protease (Promega, Madison, WI, USA) and phosphatase inhibitor cocktails (Thermo Fisher Scientific) were added for 5 min on ice, followed by centrifugation at ~14,000× *g* for 15 min. The total protein concentration was measured in duplicate using a Pierce BCA Protein Assay Kit (Thermo Fisher Scientific), and the OD was measured at 562 nm. The cell lysate was stored at −20 °C until further analysis.

### 2.5. Western Blot

Proteins (40 μg) were electrophoretically separated by 12% sodium dodecyl sulfate–polyacrylamide gel electrophoresis and transferred to polyvinylidene fluoride membranes (Millipore, Burlington, MA, USA), followed by blocking for 1 h with Odyssey Blocking Buffer (LI-COR Biosciences, Lincoln, NE, USA). The membranes were then incubated overnight at 4 °C with primary rabbit and mouse antibodies (1:1000), including antibodies against total GCN2 (Cell Signaling Technology, Danvers, MA, USA) and site-specific phosphorylated GCN2 (GCN2P) (Thr899; Biorbyt, San Francisco, CA, USA), total eIF2 and eIF2P (Ser51), α-tubulin (αTub; Cell Signaling Technology), and casein subunits α and ꞵ (ABCAM, Cambridge, UK). After five washes of 5 min each with PBS containing 0.01% Tween-20 (Bio-Rad Life Science, Hercules, CA, USA), the blots were incubated for 1 h at room temperature with goat anti-rabbit and anti-mouse secondary antibodies (1:10,000) conjugated with fluorescent dyes (IRDye 800 CW and IRDye 680, respectively; LI-COR Biosciences). Then, the blots were washed five more times as described above and scanned using the Odyssey Infrared Imaging System (LI-COR Biosciences). The signal intensities of total and phosphorylated forms of the target proteins were quantified using Odyssey application software v3.0, and the phosphorylated-to-total protein ratio was calculated as described by Arriola Apelo et al. [15]. The results were standardized as a ratio to αTub for GCN2P, total GCN2, eIF2P, total eIF2, and casein.

### 2.6. Proteomic Quantification

Samples of the RIPA cell homogenate (~200 µL) were subjected to label-free quantitative proteomics based on ion mobility-enhanced data-independent acquisition, which involves liquid chromatography–ion mobility–mass spectrometry analysis and quantification (ISOQuant software, Mainz, Germany) [16,17]. There were 1180 proteins in ≥90% of the samples for the treatments of interest (control; depletion, 2% EAA for 8 and 24 h; and RF, 100% EAA for 8 and 24 h), which were used for subsequent analyses. The quantification results of TOP3 ISOQuant were used.

### 2.7. Statistical Analysis

We evaluated the effects of various levels of EAA nutrition, fasting, and repletion on MEC viability, proliferation, and total protein expression by analyzing the differences between medians using the Kruskal–Wallis test. We also performed the nonpaired Wilcoxon rank test to compare differences among the experimental groups. For casein and total and phosphorylated GCN2 and eIF2, respectively, the same model was used but with mean values, and a Tukey test for post-hoc analysis was performed. We performed simple and multiple regression analyses to determine the relationship between eIF2P, total eIF2, GCN2P, and total GCN2 expression; the ratios of eIF2P/total eIF2 and GCN2P/total GCN2; and casein. Because of the high dimensionality of the mass spectrometry data (proteome), principal component analysis (PCA) was used to identify relationships among variables and treatments. Variables associated with principal components (PCs) were grouped according to their possible role in the PS pathways and analyzed using the nonparametric Kruskal–Wallis test. PCA analyses were followed by multiple regression to analyze the relationship between proteins from the PCs and their effects on casein synthesis. Results were considered statistically significant at *p* < 0.05 for all parametric and nonparametric analyses. The R software v3.4.2 package FactoMineR [18] and SPSS Statistics v25 (IBM Corp., Armonk, NY, USA) were used for statistical analyses.

## 3. Results

### 3.1. Viability, Proliferation, and Total Protein Concentration of MECs

EAA starvation (0% and 2%) for 8 and 24 h resulted in significant differences in the viability of MECs compared with the control cells (100% EAA; *p* < 0.05). When the cells were repleted, we also observed differences in median viability between the control and MECs exposed to 0% and 2% EAA, respectively, and refed with 100% EAA for 8 and 24 h. However, the viability ranged 97.2–99.8%, indicating an extremely low level of cell death (Figure S1). The median cell proliferation was 1.02–1.55 AU, with significant differences (*p* < 0.05) between the control cells (1.19 AU) and cells with 0% or 2% EAA depletion for 8 h (1.04 and 1.10 AU, respectively). There were also significant differences between the control cells (1.55 AU) and cells with 0% and 2% EAA depletion for 24 h that were refed for 24 h (1.34 and 1.31 AU, respectively; Figure S2). The median total protein concentration was 3158 µg/mL (range, 1962–4616 µg/mL) with no significant differences between MECs cultured with 100% EAAs and those with depleted EAAs, or those refed at different exposure times (Figure S3).

### 3.2. Casein Expression in MECs

**Figure 1:**
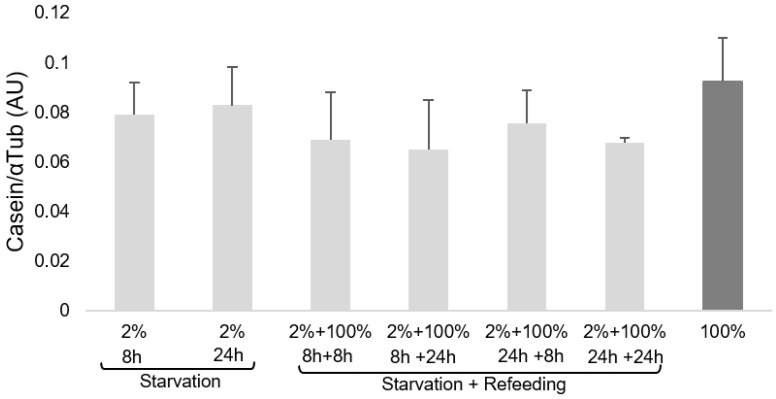
Casein expression in MECs (mean ± SD). Cells were cultured for 6 days after reaching confluence. EAAs were added at concentrations of 2% and 100% to DMEM for 8 and 24 h, followed by refeeding with 100% EAAs for 8 and 24 h. The dark gray column represents the average level of casein expression in cells not depleted (100% EAA, control) and cultured for 8, 24, and 48 h. MEC: mammary epithelial cell; SD: standard deviation; EAA: essential amino acid.

Intracellular casein levels were unaffected by treatment (mean, 0.086 ± 0.02 AU for the 100% EAA control MECs and 0.081 ± 0.002 and 0.069 ± 0.004 AU for 2% EAA starvation and starvation + repletion treatments, respectively: Figure 1).

### 3.3. Protein Expression in the GCN2/eIF2 Pathway

**Figure 2:**
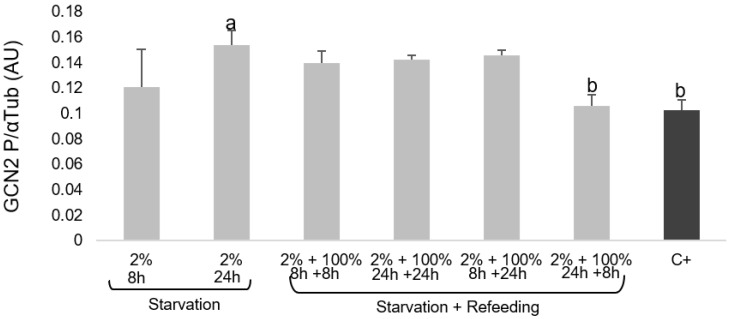
Total and phosphorylated GCN2 expression in MECs cultured for 6 days. EAAs were added at concentrations of 2% (starvation) and 100% (cells not depleted: control, C+) for 8 and 24 h, followed by refeeding with 100% EAAs for 8 and 24 h. Columns with different letters indicate significant differences. GCN2: general control nonderepressible 2; MEC: mammary epithelial cell; EAA: essential amino acid.

Under the conditions of EAA depletion, MECs showed increased GCN2P expression (*p* < 0.05) after 24 h of starvation. Repletion with 100% EAA for 8 and 24 h after exposure to 2% EAA for 8 and 24 h did not alter GCN2P expression compared with the control cells. Interestingly, RF of MECs with 100% EAA for 8 h after 24 h of starvation resulted in decreased GCN2P levels similar to those of the control cells (Figure 2).

**Figure 3:**
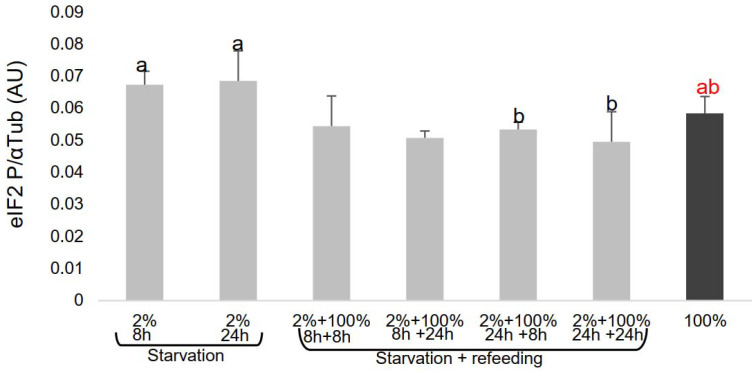
Total and phosphorylated eIF2 expression in MECs cultured for 6 days. EAAs were added at concentrations of 2% (starvation) and 100% (cells not depleted: control) for 8 and 24 h, followed by refeeding with 100% EAAs for 8 and 24 h. Columns with different letters indicate significant differences. eIF2: eukaryotic initiation factor 2; MEC: mammary epithelial cell; EAA: essential amino acid.

Similarly, the increased expression of eIF2P was observed in cells under EAA starvation compared with control cells (*p* > 0.05). Furthermore, eIF2P levels were higher when cells were starved of EAA for 8 and 24 h compared with cells refed with 100% EAA for 8 and 24 h (*p* < 0.05) (Figure 3).

Although we did not measure the ratios of GCN2 phosphorylation and eIF2 phosphorylation, a positive relationship between total GCN2 and GCN2P, and between eIF2 and eIF2P was verified (data not shown).

The relationship between casein and the expression level of individual or multiple signaling proteins was analyzed, but neither GCN2 nor GCN2P was a significant determinant. The model that best explains casein production (R2 = 0.3; *p* < 0.05) is as follows:

casein/αTub = total eIF2/αTub × 0.091 (SE 0.04) + eIF2P/αTub × 0.409 (SE 0.36)

where SE = standard error.

As expected, GCN2P and the ratio of GCN2P/total GCN2 (data not shown) in MECs depleted of EAAs was greater than that of the control and refed cells, suggesting that the MECs sensed EAA availability (Figure 2). Similarly, the rate of phosphorylation of eIF2α was higher when MECs were starved of EAAs compared with repleted cells (Figure 3).

### 3.4. MEC Proteome under Depletion and Repletion Conditions

When the complete proteome dataset was subjected to PCA, six PCs explained 79.3% of the observed total variance. Most proteins included in the six PCs (*n* = 30) were transcription factors, translation factors, or peptidases required for PS, and 21 of the 30 proteins were related to translation (initiation and elongation factors). Thus, according to PCA, the primary cell function affected by the treatments was PS. This is unsurprising given that our treatments solely involved manipulating the EAA supply to MECs. However, if global protein translation is affected by these factors, changes in the concentrations of many intracellular proteins can be anticipated.

With respect to treatments, nonparametric analyses of changes in the concentrations of the 30 proteins according to PCs that they belonged to indicated that EAA starvation and RF led to the differential expression of 16 proteins: G1 to S phase transition protein 1 (GSPT1), casein kinase 2 alpha 1 (CSNK2A1), eIF1, eIF1A, eIF2S1, eIF3F, eIF5A, eIF6, eIF4A1, eEF1B, eEF1D, eEF1G, peptidase D (PEPD), peptidylprolyl isomerase (PPI) A, PPIB, and FKBP prolyl isomerase 3 (FKBP3), compared with the control (shown in bold in Table 1, quantification in Table S1).

We grouped and analyzed the 30 proteins according to their possible role in PS pathways. Groupings were defined according to cell cycle, proliferation, translation (initiation and elongation factors), and posttranslational processes, resulting in six categories (Table 1). In each functional group, ≥50% of the proteins were affected by the treatments, except for group 4 (initiation factors turning on translation), in which only eIF4A1 was affected.

Our multiple regression analysis included casein as a dependent variable explained by the 30 proteins of the six different PCs in six models. Three of these models were significant (*p* < 0.05), explaining 65%, 79%, and 92% of the observed variance in casein levels. The significant predictor proteins included in these three models (*p* < 0.05) were PEPD, eIF1A, eIF5A, eEF1G, and eEF1D.

### 3.5. Factors Regulating PS

#### 3.5.1. Responses to EAA Depletion for 8 and 24 h

Cells subjected to 8 h of starvation showed reduced eIF3F expression compared with the cells treated with 100% EAA (*p* < 0.05). Furthermore, FKBP3, a PPI posttranslational protein thought to be involved in cell proliferation, cell cycle progression, cell survival, and casein synthesis, was increased. When MECs were subjected to 24 h of EAA depletion, the expression of two initiation factors (eIF5A and eIF4A1) and one elongation factor (eEF1G) was inhibited. These changes suggest a reduction in translation rates, which is expected to conserve EAAs. Depletion also stimulated the production of two PPIs (PPIA and PPIB) (*p* < 0.05; Table S1: quantifications, see Supplementary Materials).

#### 3.5.2. Responses to Repletion after 8 h of EAA Depletion

Repletion for 8 or 24 h after 8 h of EAA depletion significantly inhibited FKBP3 (Table 1; *p* < 0.05). CSNK2A1 was also inhibited by 24 h of repletion. eIF1 and eIF3F expression increased in response to 8 h of repletion, whereas eIF2S1 was inhibited. Repletion for 24 h reversed the responses of eIF1 and eIF2S1 and negated the response of eIF3F. The longer repletion time also stimulated eIF6 expression and inhibited eIF4A1 expression. These proteins are all involved in regulating translation, reflecting the critical role that translational regulation plays in response to EAA supply. Protein eIF4A1 was the only factor inhibited after 24 h of depletion and 8 and 24 h of repletion, suggesting that cells recovered and initiated translation via different pathways, as implied by the initiation factors in group 3 (Table 1).

#### 3.5.3. Responses to Repletion after 24 h of EAA Depletion

Following repletion for 8 h after 24 h of starvation, the expression of three proteins (GSPT1, eIF1A, and PEPD) was inhibited, and that of six proteins (three initiation factors, two elongation factors, and PPIB) was induced. However, when MECs were repleted for 24 h, the level of only one protein increased (GSPT1), whereas seven proteins were inhibited. The latter belonged to the initiation, elongation, and posttranscriptional groups (Table 1), suggesting that a wide range of alternate pathways may be involved when MECs are subjected to EAA fasting.

In addition to the 30 proteins (Table 1) related to the GCN2/eIF2 pathway, 10 other proteins previously reported to be important in mammary gland development and milk production were found in the proteome results. Nine of them (actin-related proteins, annexins, gelsolin, heat shock proteins, integrins, phosphoglycerate mutase, proteasome subunit beta, serpins, and S100 proteins) were unaffected by starvation and RF treatments. The only regulated protein was vimentin: control + 100% EAA showed higher vimentin expression compared with cells depleted to 2% EAA for 24 h and repleted for 8 h (*p* = 0.029) or 24 h (*p* = 0.006). However, regression of the proliferation rate on vimentin levels revealed that vimentin did not significantly affect MEC proliferation (intercept = 1.279, *p* < 0.05; vimentin coefficient = −2.751 × 10^−7^; *p* = 0.905).

## 4. Discussion

### 4.1. Lactocyte (MEC) Model Validity

Among the keratins that we identified (see Section 2.1), cytokeratin 18 (acidic type) and cytokeratins 7, 8, and 9 (basic type) indicated that the cells were epithelial in nature and all of bovine (Bos taurus) origin. The expression of both keratins and vimentins in the monolayers of bovine MECs has been reported [19]. The presence of these markers of bovine epithelial cells and casein confirm that the cells were bovine MECs. Factors such as insulin, growth hormone, hydrocortisone, and epidermal growth factor can influence the rates of metabolism, cell cycle progression, milk PS, cell viability, and proliferation [13]. However, none of these factors affected cell viability (Figure S1).

The treatments were not so severe as to cause permanent cell damage. We suggest that in live mammary glands, an external supplementation of EAA would result in cell adaptation and the maintenance of viability and proliferation necessary to guarantee sustained milk production. Aminogenic and other substrates supplemented to dairy cows can modify the profile of AA available for mammary metabolism and determine the level of mammary EAA uptake [7].

### 4.2. Casein and Related Proteins

Although detectable through immunoblotting, casein was not observed in the proteome data, suggesting that the expression levels were relatively low or that the casein peptides generated were poorly ionized by mass spectrometry. Our findings (Figure 1) indicate that casein synthesis could be one of the homeorhetic processes of the homeostatic capacity of MECs proposed in our hypothesis to overcome nutrient starvation by the GCN2/eIF2 pathway [20]. We suggest that different levels of proteins found in our multiple regression analysis are related to variations in the proteins orchestrating the maintenance of casein production under stress conditions. The response would be to continue the eIF2 downstream pathway with differential protein expression, possibly including eIF1A, eIF5A, eEF1D, and PEPD, to regulate milk PS. This could contribute to characterizing the role of eIF2α in the adaptive AA response and in response to other stressors, and could reveal greater insight into its function in milk synthesis [2]. Further studies are warranted to better understand the roles and interactions of these predictors in regulating casein synthesis.

### 4.3. Relationship between the GCN2/eIF2 Pathway and MEC Protein Expression (Proteome)

Proteins eIF2S3 and eIF2S1 were represented in PC1 and PC2, respectively, indicating significant correlations among these factors and the overall variance in protein expression. The loading was negative for eIF2S3 and positive for eIF2S1. The latter was consistent with the stimulatory role of eIF2α, with respect to mRNA translation [21]. Conversely, the negative correlation of eIF2S3 with protein expression was contrary to our expectations; however, the eIF2 cycle and its kinases can inhibit global translation and simultaneously stimulate gene-specific translation [21]. 

The factors identified to stimulate translation as a part of the derepression mechanism include eIF3 and the eIF4 family members eIF4A1, eIF4A2, and eIF4A3 [22] and positive regulated protein expression [3]. In addition to the putative positive effects of the increased expression of the eIF class, eEF2, elongation factors Tu and Ts, eEF1α P1 and P2, eEF1δ, and eEF1β were all positively correlated with total protein expression. Generally, these should all be positively correlated with the rates of translation; however, eEF1γ, eIF1A, and eIF5A were negatively correlated with overall protein expression, which is inconsistent with their roles in elongation and initiation [23]. GSPT1 (eukaryotic release factor 3a) expression was also negatively associated with overall protein expression, which is consistent with the concept of repressed differentiation function during cell replication [24,25]. GSPT1 and cell division control protein 42, which are checkpoint proteins, showed reduced expression—this tends to maintain proliferation without affecting viability and total PS, as GSTP1 is also a classic detoxification enzyme that has been implicated in resistance to apoptosis initiated by a variety of stimuli, which includes the deprivation of growth factors [26] or, as in our case, of EAA.

Similarly, the expression of STAT 3 and STAT1 was negatively correlated with protein expression. These transcription factors play a role in the MEC response to nutritional stress contributing to increased amino acid availability [8]. Moreover, STAT1 and STAT3 were not significantly affected by the EAA treatments. STAT5, a key factor for casein synthesis and a central determinant of mammary gland development and function [27], was unaffected by the treatments in our study. During lactation, STAT5 governs milk protein gene expression and contributes to cell survival [28,29]. These findings suggest that the EAA concentrations and/or depletion and repletion exposure times regulate different signaling pathways to maintain casein expression.

A deficiency of all three branched-chain AAs (isoleucine, leucine, and valine) may impair milk protein yield by deactivating mTORC1-mediated upregulation of eIF2B and eIF2A [30]. This is consistent with our finding that the initiation factors eIF2A and eIF2B were not significantly affected by the EAA treatments. Translation initiation and elongation factors have been suggested as potential targets for the regulation of milk production and, thus, potentially milk PS, possibly via the mTOR pathway [31]. Although we did not measure mTOR expression in our study, EAA depletion resulted in the decreased expression of three initiation factors (eIF3F, eIF4A1, and eIF5A) and one elongation factor (eEF1G) implicated in casein synthesis [5,6]. This downregulation did not affect casein levels, perhaps because of alternative regulation factors, such as those upregulated when MECs were repleted (eIF1, eIF2S1, eIF6, eEF1B, and eEF1D). 

Proteins, such as PEPD, PPIs (PPIA, PPIB, PPIC, and FKBP3), and CSNK2A1, participate in posttranscriptional mechanisms and are affected by EAA treatments. PEPD is essential for catalyzing the last step of the degradation of procollagen, collagen, and other peptides into free AAs to be used for cellular growth. PPIs accelerate protein folding. CSNK2A1 is a serine/threonine protein kinase that phosphorylates acidic proteins, such as casein, and is also thought to be involved in regulating cell proliferation, differentiation, and apoptosis [29]. 

We hypothesized that exogenously treating bovine MECs with varying concentrations of EAAs for different exposure times would affect the cellular proteome, including the signaling proteins regulating PS. Our findings support, in part, this hypothesis of an intricate mechanism of PS regulation by EAA profile changes. Recently, Cant et al. [32] reviewed the literature pertaining to early signaling events and their long-term consequences on milk protein yield in mammary cells in vitro and in vivo. They suggested that the signaling networks did not account for long-term changes in milk protein yields. We did not measure and analyze milk yields because this was outside the scope of our study. Thus, further studies are necessary to better understand long-term changes in EAA supply, signaling, and milk protein yields.

Among the 10 proteins found in the proteome that are important for mammary gland development and milk production, only vimentin was regulated after in vitro challenges. Based on the role of vimentin [33] the treatment effects observed in our study suggest that EAA starvation affected cell proliferation (Figure S2). However, the regression results indicated that such effects on the MEC proliferation rate were not significant, and that vimentin did not act alone, as expected, to regulate proliferation.

In vivo supplementation of specific AAs (as a rumen by-pass protein supply [7]) that would exert the most influence on pathways controlling eIFs and eEFs should enable a reduction in crude protein supply to dairy animals, leading to increased postabsorptive efficiency and reduced N excretion [6]. Our in vitro findings and those of Yoder et al. [34] can contribute to achieve this key dairy management goal. 

MECs facing EAA stress maintained protein and casein synthesis processes by adapting molecular mechanisms, such as those involving eIFs, eEFs, and transcription factors, to achieve PS homeostasis; this correlates with similar previous findings [35]. Nevertheless, further in vivo studies are warranted to optimize protein supplies to dairy cows.

## 5. Conclusions

We confirmed that several EAAs regulate the rate of casein synthesis by altering the phosphorylation states of intracellular signaling proteins affecting the activity of initiation (eIFs) and elongation (eEFs) factors. 

As a model of low protein supply, our MECs under EAA starvation maintained protein and casein synthesis processes by adapting molecular mechanisms, such as those involving eIFs, eEFs, and transcription factors. This supports transcriptional adaptation (homeorhesis) by MECs facing EAA stress to achieve PS homeostasis.

## Figures and Tables

**Table 1:** Proteins (*n* = 30) related to protein synthesis in the MEC model and effects of different treatments (Trt.: depletion with 8 and 24 h with 2% EAA; repletion with 100% EAA for 8 and 24 h after 8 and 24 h of depletion) on protein expression. Significant differences in protein expression (*p* < 0.05) are shown in bold (*n* = 16). Arrows (up or down) indicate the effect of treatment on protein concentration compared with the control (100% EAAs). MEC: mammary epithelial cell; EAA: essential amino acid; Trt: treatment; GSPT1: G1 to S phase transition 1; CSNK2A1: casein kinase 2 alpha 1; CDCP42: cell division control protein 42; STAT: signal transducer and activator of transcription; eIF: eukaryotic initiation factor; eEF: eukaryotic elongation factor; PEPD: peptidase D; PPI: peptidylprolyl isomerase; TPP1: tripeptidyl-peptidase 1; FKBP3: FKBP prolyl isomerase 3.

Protein Group	Protein/Trt	2% EAA 8 h	2% EAA 24 h	2% EAA 8 + 8 h RF	2% EAA 8 + 24 h RF	2% EAA 24 + 8 h RF	2% EAA 24+ 24 h RF
1. Cell proliferation/cell cycle progression	**GSPT1**					↓	↑
	**CSNK2A1**				↓		
	CDCP42						
2. Transcription factors	STAT1						
	STAT3						
3. Initiation factors that turn off translation or are turned off by eIF2	**eIF1**			↑	↓	↑	↓
	**eIF1A**					↓	
	**eIF2S1**			↓	↑	↑	↓
	eIF2S3						
	eIF3A						
	eIF3C						
	**eIF3F**	↓		↑			
	eIF3J						
	**eIF5A**		↓				
	**eIF6**				↑	↑	
4. Initiation factors that turn on translation	eIF3						
	eIF4						
	**eIF4A1**		↓		↓		↓
	eIF4A2						
	eIF4A3						
5. Elongation factors	eEF1A2						
	**eEF1B**					↑	↓
	**eEF1D**					↑	↓
	**eEF1G**		↓				
	eEF2						
6. Posttranslational proteins	**PEPD**					↓	
	**PPIA**		↑				↓
	**PPIB**		↑			↑	↓
	**CSNK2A1**				↓		
	TPP1						
	**FKBP3**	↑		↓	↓		

## Data Availability

The data presented in this study are available on request from the corresponding author.

## Supplementary Materials

The following supporting information can be downloaded at: https://www.mdpi.com/article/10.3390/cimb44030071/s1.
