# Supplementary material for: Effects of Essential Amino Acid Deficiency on General Control Nonderepressible 2/Eukaryotic Initiation Factor 2 Signaling and Proteomic Changes in Primary Bovine Mammary Epithelial Cells
Source: Curr Issues Mol Biol. 2022 Feb 25;44(3):1075–86. doi: 10.3390/cimb44030071 (PMC8947524; doi:10.3390/cimb44030071)
Supplement: Supplementary file 1 [file cimb-44-00071-s001.zip › cimb-1556287-supplementary.pdf]

Supplementary Materials

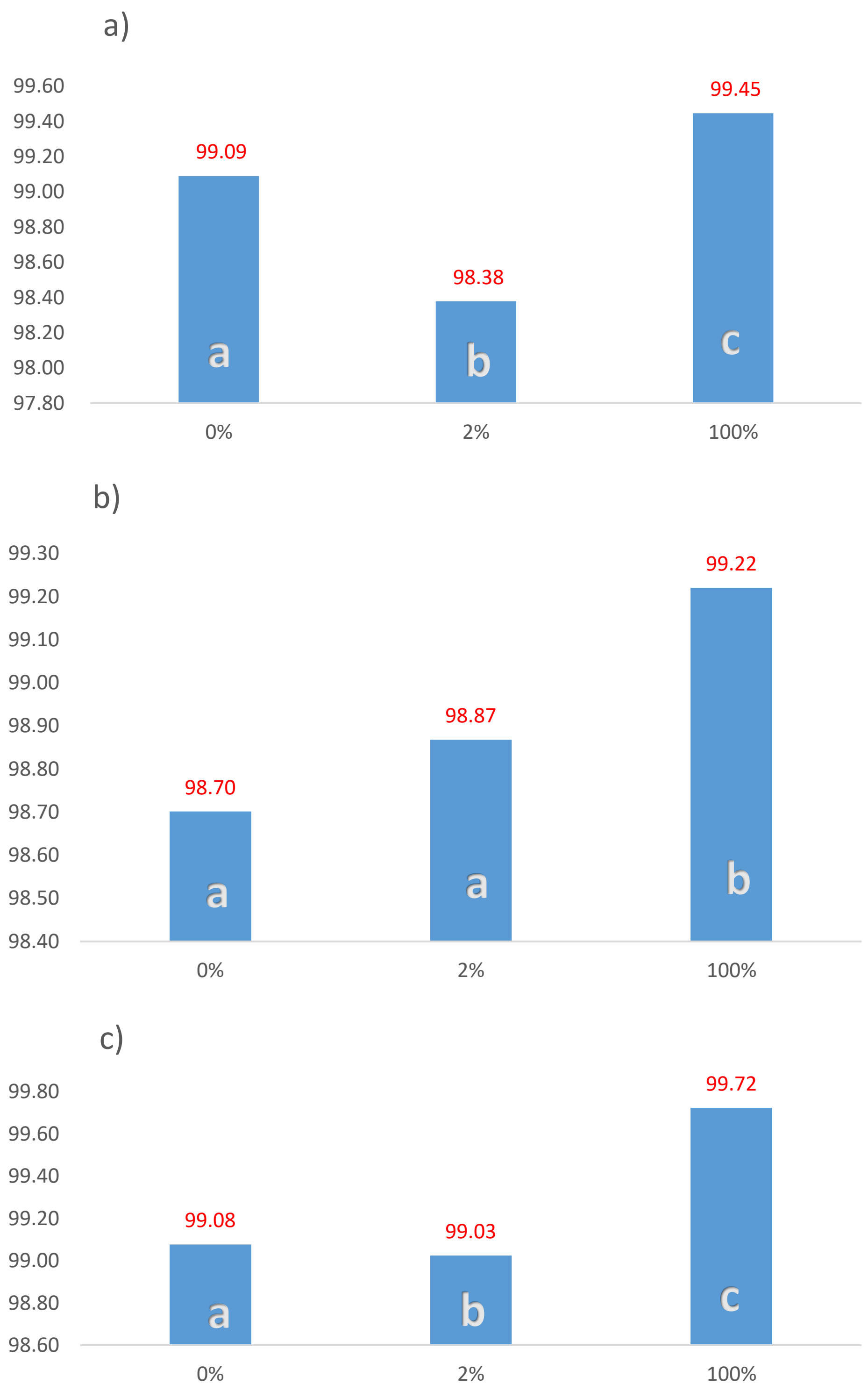

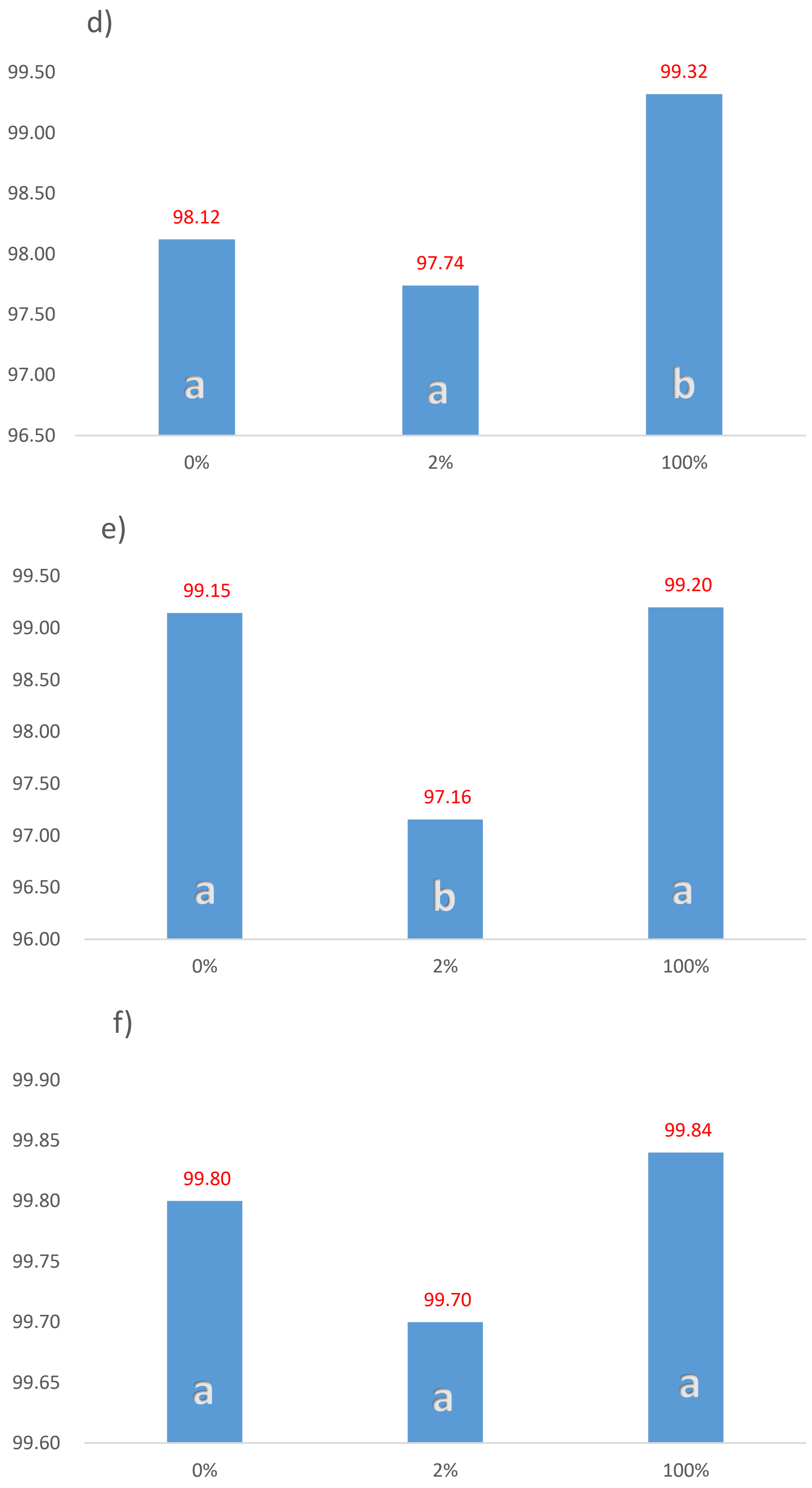

Figure S1. Cell viability (%) of four different experiments (medians) of MEC in culture for 6 days after confluence. Essential amino acids (EAA) were added at doses of 0%, 2%, and 100% of complete DMEM concentrations for 8 h & 24 h and repletion (R) was done with 100% EAA for 8 h & 24 h. (**a**) 8 h depletion (D): 0% and 2% EAA. (**b**) 24 h D. (**c**) 8 h D + 8 h R. (**d**) 8 h D + 24 h R. (**e**) 24 h D + 8 h R.

(**f**) 24 h D + 24 h R. In red, min. and max. values. Columns with different letters depict significant differences ($p < 0.05$).

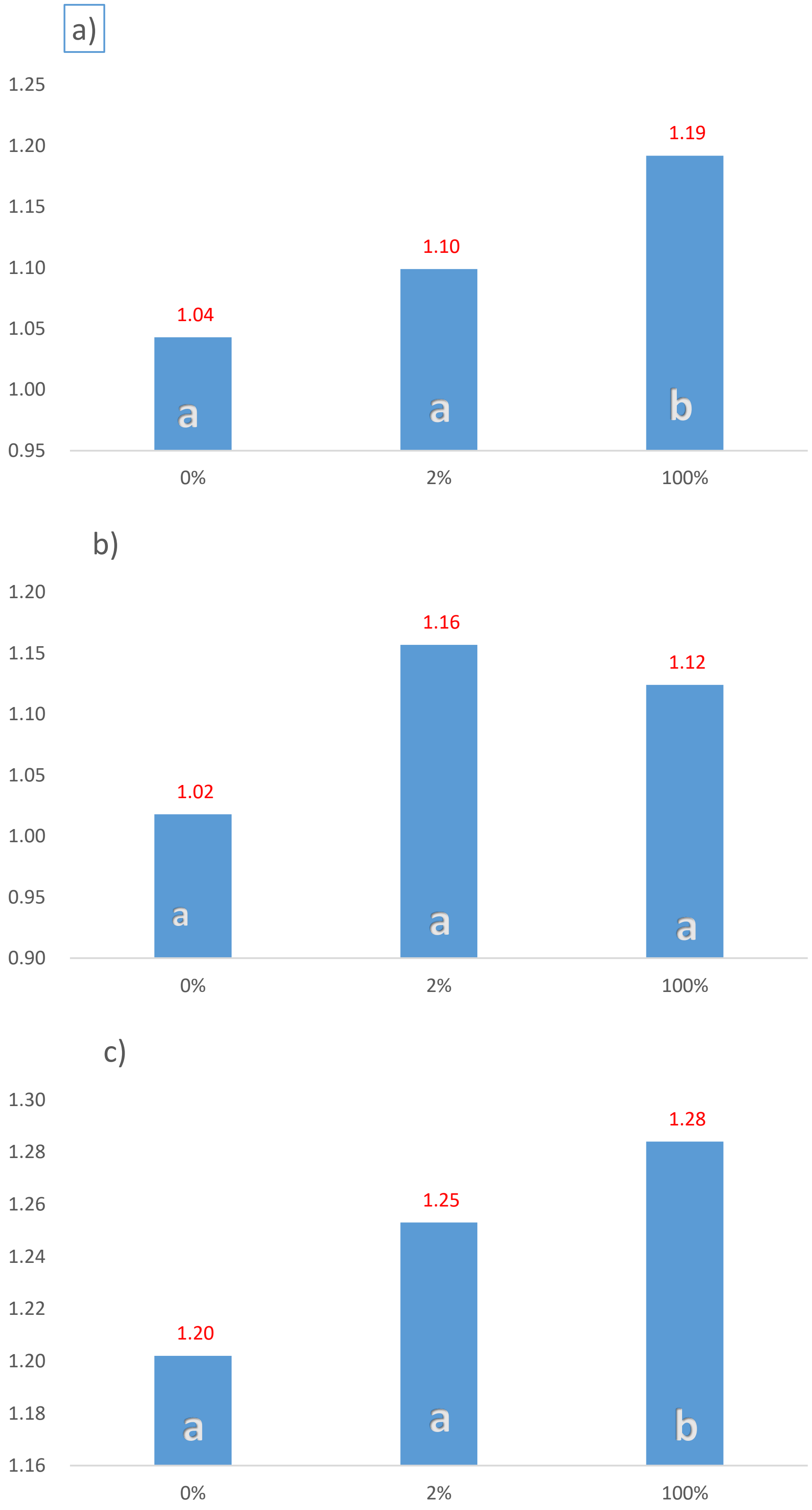

d)

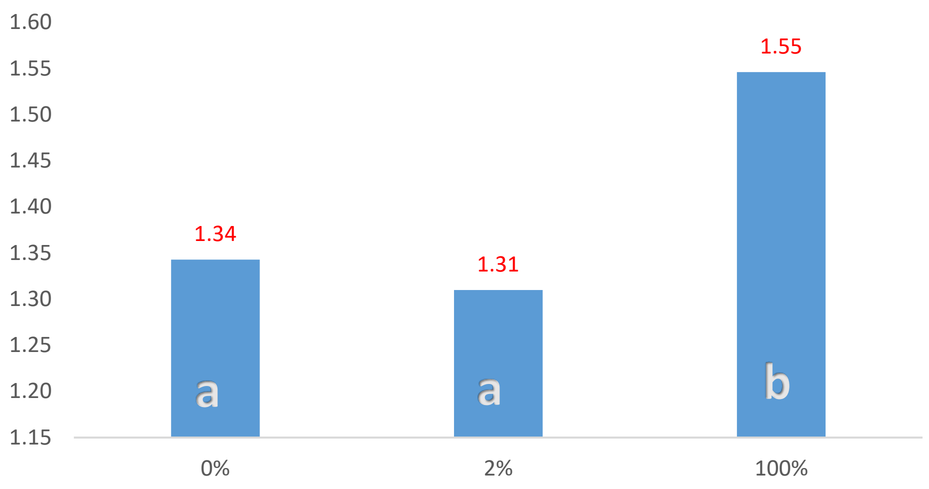

Figure S2. Cell proliferation (optical density, OD) of four different experiments (medians) of MEC in culture for 6 days after confluence. Essential amino acids (EAA) were added at doses of 0%, 2%, and 100% of complete DMEM concentrations for 12 h & 24 h and repletion (R) was done with 100% EAA for 12 h & 24 h. (**a**) 12 h depletion (D): 0% and 2% EAA. (**b**) 24 h D. (**c**) 12 h D + 12 h R. (**d**) 24 h D + 24 h R. In red, min. and max. values. Columns with different letters depict significant differences ($p < 0.05$).

a)

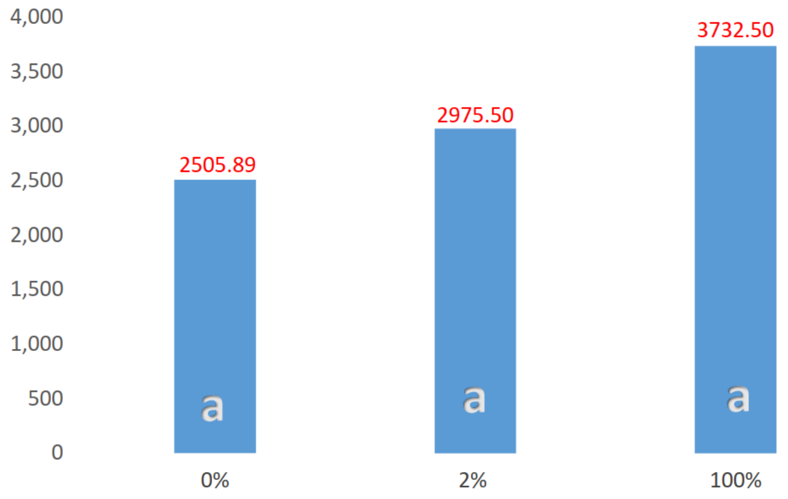

b)

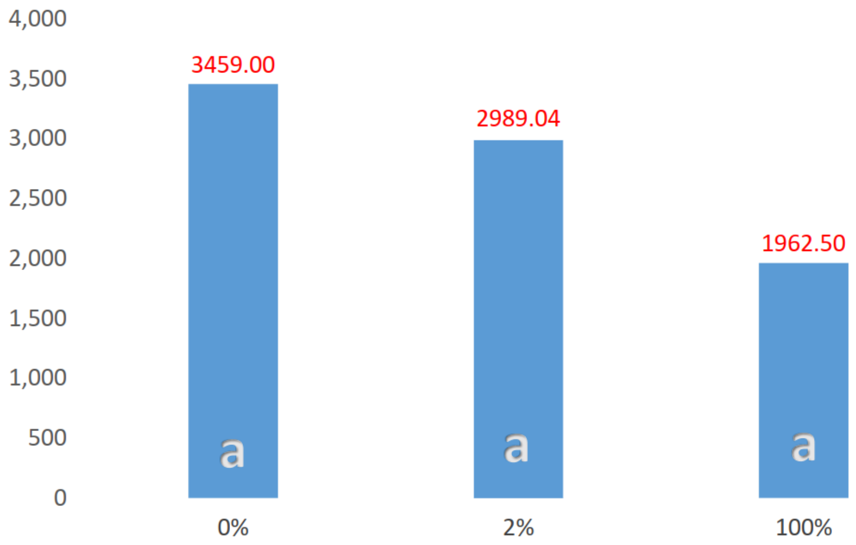

4,000
3,500
3,000
2,500
2,000
1,500
1,000
500
0
3459.00
2989.04
1962.50
a
a
a
0%
2%
100%

c)

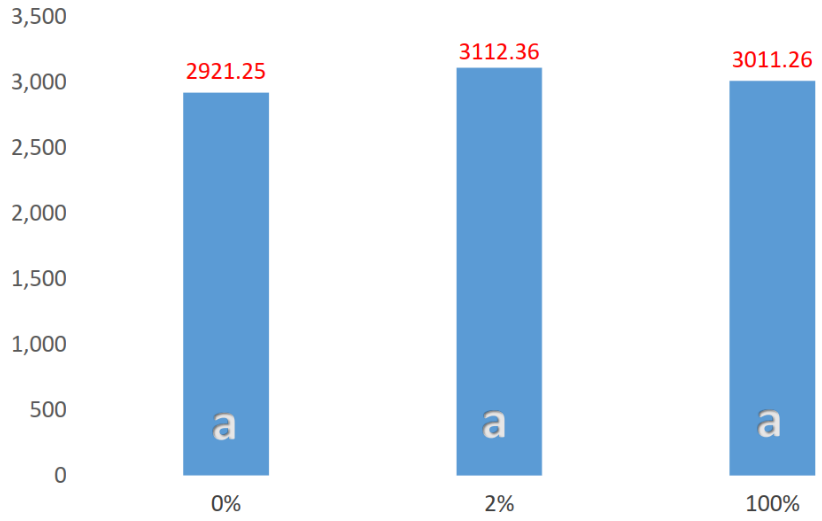

3,500
3,000
2,500
2,000
1,500
1,000
500
0
2921.25
3112.36
3011.26
a
a
a
0%
2%
100%

d)

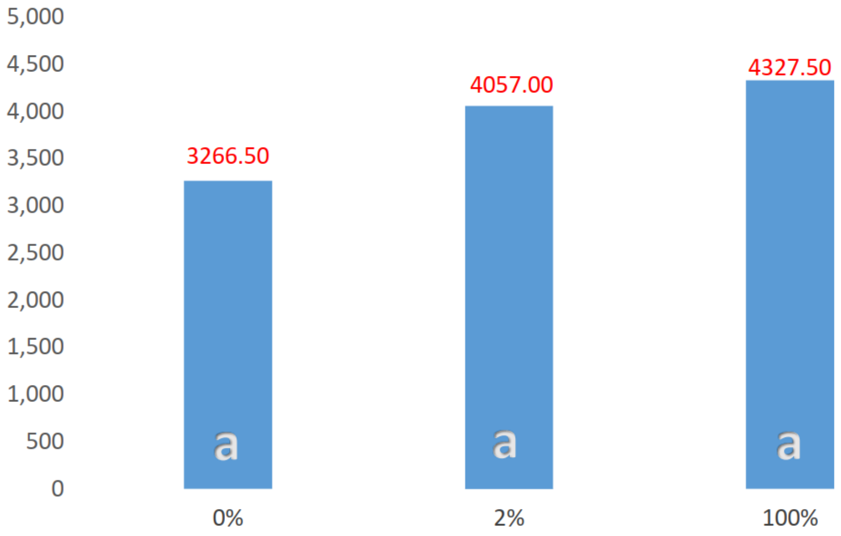

5,000
4,500
4,000
3,500
3,000
2,500
2,000
1,500
1,000
500
0
3266.50
4057.00
4327.50
a
a
a
0%
2%
100%

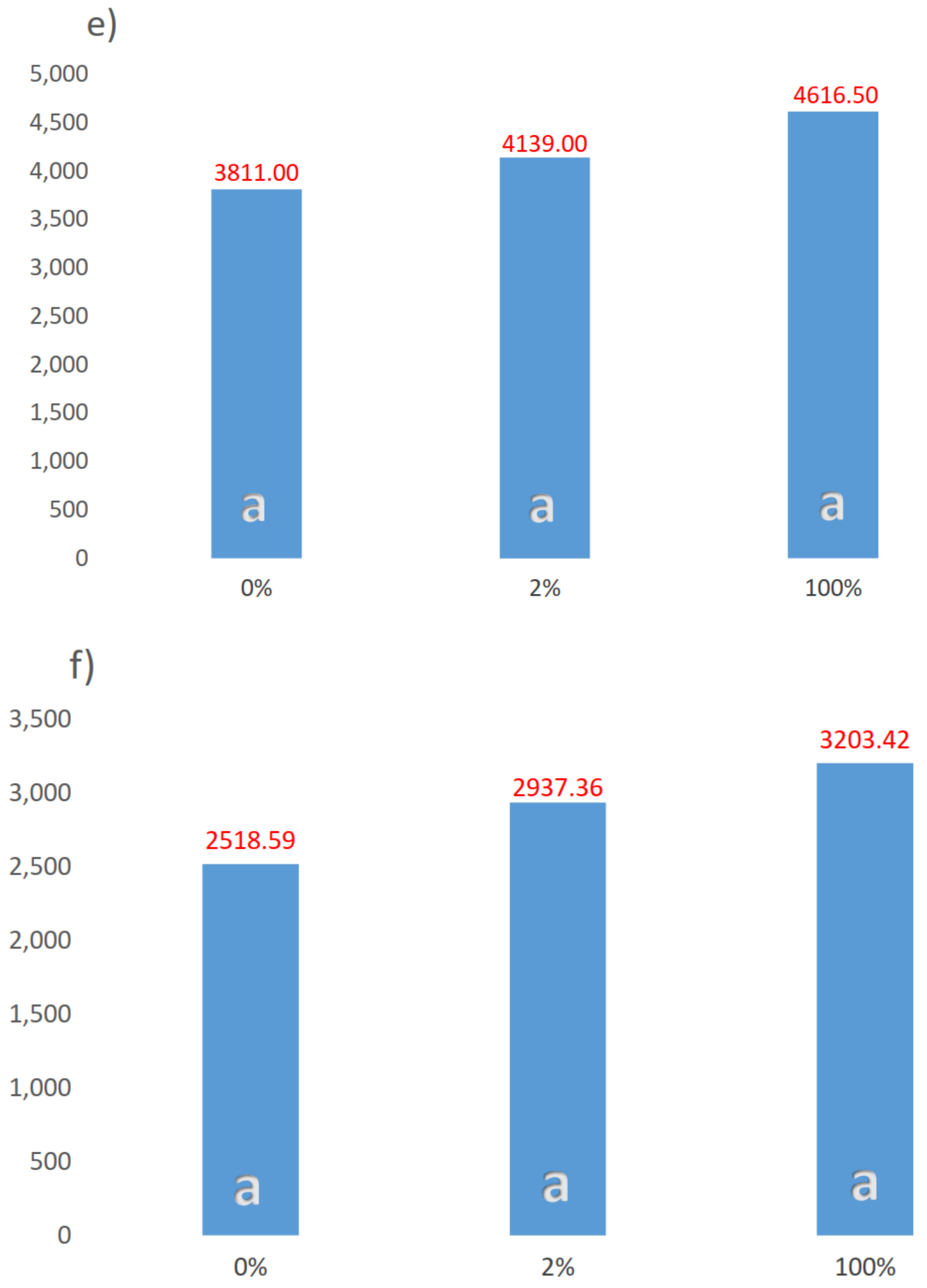

Figure S3. Total protein (ug/mL) of four different experiments (medians) of MEC in culture for 6 days after confluence. Essential amino acids (EAA) were added at doses of 0%, 2%, and 100% of complete DMEM concentrations for 8 h & 24 h and repletion (R) was done with 100% EAA for 8 h & 24 h. (**a**) 8 h depletion (D): 0% and 2% EAA. (**b**) 24 h D. (**c**) 8 h D + 8 h R. (**d**) 8 h D + 24 h R. (**e**) 24 h D + 8 h R. (**f**) 24 h D + 24 h R. In red, min. and max. values. Columns with different letters depict significant differences ($p < 0.05$).

Table S1. Proteins (n = 16) with significant different expression ($p < 0.05$) in MEC. Protein absolute concentration (TOP3 ISOQuant) indicate the effect of treatment compared to the control (bold). MEC: mammary epithelial cell; EAA: essential amino acid; Trt: treatment; GSPT1: G1 to S phase transition 1; CSNK2A1: casein kinase 2 alpha 1; CDCP42: cell division control protein 42; STAT: signal transducer and activator of transcription; eIF: eukaryotic initiation factor; eEF: eukaryotic elongation factor; PEPD: peptidase D; PPI: peptidylprolyl isomerase; TPP1: tripeptidyl-peptidase 1; FKBP3: FKBP prolyl isomerase 3.

	Protein/Trt.	N	Mean	Standard deviation
1. GSPT1	1. 2%EAA-8h	6	61518,4167	6529,16468
	2. 2%EAA-24h	6	60787,5417	2146,97394
	3. 2%EAA-8h+8h RF	6	61207,0000	7270,23361
	4. 2%EAA-24h+24h RF	6	**72657,7917**	7531,17518
	5. 2%EAA-8h+24h RF	6	**60340,1250**	2947,36459
	6. 2%EAA-24h+8h RF	6	**55079,8333**	1936,64809
	7.Control (100% EAA)	6	**63926,4167**	3753,98870
2. CSNK2A1	1. 2%EAA-8h	6	13881,7917	2990,12349
	2. 2%EAA-24h	6	15396,9444	2563,18171
	3. 2%EAA-8h+8h RF	6	14603,4583	3275,45687
	4. 2%EAA-24h+24h RF	6	15499,4861	1519,24471
	5. 2%EAA-8h+24h RF	6	**11568,3750**	848,05662
	6. 2%EAA-24h+8h RF	6	14307,3056	3224,96553
	7.Control (100% EAA)	6	**16327,7778**	916,32417
3. eIF1	1. 2%EAA-8h	6	19827,2500	5736,12375
	2. 2%EAA-24h	6	22331,4444	4293,68913
	3. 2%EAA-8h+8h RF	6	**32237,9722**	5416,49531
	4. 2%EAA-24h+24h RF	6	**14433,1389**	5147,10302
	5. 2%EAA-8h+24h RF	6	**15223,8611**	2668,14557
	6. 2%EAA-24h+8h RF	6	**30452,5278**	4410,63844
	7.Control (100% EAA)	6	**18304,9722**	3623,56032
4. eIF1A	1. 2%EAA-8h	6	14696,1667	1272,23067
	2. 2%EAA-24h	6	13527,0000	752,63583
	3. 2%EAA-8h+8h RF	6	14602,1667	1587,77702
	4. 2%EAA-24h+24h RF	6	13409,5833	1507,28572
	5. 2%EAA-8h+24h RF	6	16902,1667	746,07015
	6. 2%EAA-24h+8h RF	6	**12992,3333**	1015,04210
	7.Control (100% EAA)	6	**16535,0833**	639,64869
5. eIF2S1	1. 2%EAA-8h	6	26848,4444	2325,43013
	2. 2%EAA-24h	6	23297,0000	1025,34339
	3. 2%EAA-8h+8h RF	6	**22371,4444**	1616,11326
	4. 2%EAA-24h+24h RF	6	**21028,6944**	673,74108
	5. 2%EAA-8h+24h RF	6	**26851,5000**	2071,51642
	6. 2%EAA-24h+8h RF	6	25486,7222	1498,41994
	7.Control (100% EAA)	6	**25548,2222**	1715,58047
6. eIF3SF	1. 2%EAA-8h	6	**15437,7222**	2149,27462
	2. 2%EAA-24h	6	14467,0000	1862,89570
	3. 2%EAA-8h+8h RF	6	**21650,1111**	1998,36541
	4. 2%EAA-24h+24h RF	6	16530,6667	3023,74825
	5. 2%EAA-8h+24h RF	6	16371,0000	1837,65537
	6. 2%EAA-24h+8h RF	6	17482,7778	2181,43182
	7.Control (100% EAA)	6	**18158,3889**	544,41558
7. eIF5A	1. 2%EAA-8h	6	104098,0555	36384,26414
	2. 2%EAA-24h	6	**57147,8333**	3629,65303
	3. 2%EAA-8h+8h RF	6	146990,9444	9785,97573

	4. 2%EAA-24h+24h RF	6	107660,5000	22306,12224
	5. 2%EAA-8h+24h RF	6	123902,2778	7681,66169
	6. 2%EAA-24h+8h RF	6	72752,0556	21310,16172
	7.Control (100% EAA)	6	**138229,3333**	6202,97205
	1. 2%EAA-8h	6	18052,0556	1792,68203
	2. 2%EAA-24h	6	16811,9444	1160,45733
	3. 2%EAA-8h+8h RF	6	15169,8333	3681,12403
8. eIF6	4. 2%EAA-24h+24h RF	6	13314,5556	3934,47769
	5. 2%EAA-8h+24h RF	6	**20284,5000**	1929,52217
	6. 2%EAA-24h+8h RF	6	**20510,4722**	868,06696
	7.Control (100% EAA)	6	**14069,2222**	2343,10766
	1. 2%EAA-8h	6	93665,0556	3732,68439
	2. 2%EAA-24h	6	**82193,2778**	6141,14518
	3. 2%EAA-8h+8h RF	6	94987,2222	1887,91785
9. eIF4AI	4. 2%EAA-24h+24h RF	6	**73982,7778**	5905,37881
	5. 2%EAA-8h+24h RF	6	**84060,1111**	4319,57867
	6. 2%EAA-24h+8h RF	6	93161,0556	6695,84797
	7.Control (100% EAA)	6	**98839,0000**	8292,30387
	1. 2%EAA-8h	6	110513,7778	6074,77321
	2. 2%EAA-24h	6	108611,2778	4915,68838
	3. 2%EAA-8h+8h RF	6	111411,6111	2321,30963
10. eEF1B	4. 2%EAA-24h+24h RF	6	**81977,3333**	10697,50225
	5. 2%EAA-8h+24h RF	6	103035,5000	12531,71038
	6. 2%EAA-24h+8h RF	6	**118671,5556**	4299,26868
	7.Control (100% EAA)	6	**102481,6667**	4458,50327
	1. 2%EAA-8h	6	89388,9444	4376,28754
	2. 2%EAA-24h	6	86975,0000	6976,83018
	3. 2%EAA-8h+8h RF	6	86355,8889	1240,22273
11. eEF1D	4. 2%EAA-24h+24h RF	6	**74357,8333**	3696,89552
	5. 2%EAA-8h+24h RF	6	93398,1111	2415,30771
	6. 2%EAA-24h+8h RF	6	**97511,3333**	3952,54807
	7.Control (100% EAA)	6	**82956,5556**	3606,90690
	1. 2%EAA-8h	6	40553,2500	8263,34519
	2. 2%EAA-24h	6	**33598,3333**	1252,23874
	3. 2%EAA-8h+8h RF	6	46484,7639	1751,70111
12. eEF1G	4. 2%EAA-24h+24h RF	6	36683,6111	3787,42059
	5. 2%EAA-8h+24h RF	6	48050,8333	6886,87825
	6. 2%EAA-24h+8h RF	6	36544,9722	3222,24806
	7.Control (100% EAA)	6	**50804,3611**	2987,20223
	1. 2%EAA-8h	6	10798,6667	1911,22031
	2. 2%EAA-24h	6	10929,8333	1046,08497
	3. 2%EAA-8h+8h RF	6	10375,0000	688,07441
13. PEPD	4. 2%EAA-24h+24h RF	6	10811,6667	880,03470
	5. 2%EAA-8h+24h RF	6	9811,66667	559,387045
	6. 2%EAA-24h+8h RF	6	**8992,66667**	518,63886
	7.Control (100% EAA)	6	**12212,6667**	681,57983
	1. 2%EAA-8h	6	394485,7778	22825,15368
	2. 2%EAA-24h	6	**392097,5556**	5061,23107
	3. 2%EAA-8h+8h RF	6	373801,2778	7786,79842
14. PPIA	4. 2%EAA-24h+24h RF	6	**333802,8333**	40100,08853
	5. 2%EAA-8h+24h RF	6	370872,8889	21251,29234
	6. 2%EAA-24h+8h RF	6	384339,5556	4009,78143
	7.Control (100% EAA)	6	**385431,8889**	6563,67064
	1. 2%EAA-8h	6	85435,0556	4400,26962
15. PPIB	2. 2%EAA-24h	6	**87978,9444**	7081,97468
	3. 2%EAA-8h+8h RF	6	73779,4444	613,56060

	4. 2%EAA-24h+24h RF	6	**63774,6667**	3299,87131
	5. 2%EAA-8h+24h RF	6	**82999,8889**	3151,96440
	6. 2%EAA-24h+8h RF	6	90479,4444	2467,49988
	7.Control (100% EAA)	6	**72142,7778**	4371,20316
16. FKBP3	1. 2%EAA-8h	6	**11494,8333**	669,45008
	2. 2%EAA-24h	6	10824,3333	589,56952
	3. 2%EAA-8h+8h RF	6	**10020,4444**	701,17595
	4. 2%EAA-24h+24h RF	6	10630,5000	578,88190
	5. 2%EAA-8h+24h RF	6	**11791,7778**	171,26596
	6. 2%EAA-24h+8h RF	6	10803,8889	304,59369
	7.Control (100% EAA)	6	**10783,3889**	550,96301
